# Challenges in Laboratory Diagnosis of Malaria in a Low-Resource Country at Tertiary Care in Eastern Nepal: A Comparative Study of Conventional vs. Molecular Methodologies

**DOI:** 10.1155/2021/3811318

**Published:** 2021-12-28

**Authors:** Pragyan Dahal, Basudha Khanal, Keshav Rai, Vivek Kattel, Satish Yadav, Narayan Raj Bhattarai

**Affiliations:** ^1^Department of Microbiology, B. P. Koirala Institute of Health Sciences, Dharan, Nepal; ^2^Department of Internal Medicine, B. P. Koirala Institute of Health Sciences, Dharan, Nepal; ^3^Department of Pediatrics, B. P. Koirala Institute of Health Sciences, Dharan, Nepal

## Abstract

For ongoing malaria elimination programmes, available methods such as microscopy and rapid diagnostic tests (RDTs) cannot detect all malaria cases in acute febrile illness. These methods are entirely dependent on the course of infection, parasite load, and skilled technical resources. Our study objectives were to estimate the performance of light microscopy and a RDT as well as real-time PCR for the detection of the *Plasmodium* parasite. Altogether, 52 blood samples collected from patients with acute febrile illness were tested by microscopy, RDT, and real-time PCR. The results were compared in terms of sensitivity and specificity. Microscopy detected the malaria parasite in 5.8% of the blood samples whereas 13.5% were detected by the RDT and 27% by real-time PCR. Considering real-time PCR as the gold standard method, microscopy had a sensitivity of 21.4% and a specificity of 100%, and the RDT had a sensitivity of 28.6% and a specificity of 92.1%. Microscopy together with the RDT successfully detected malaria positive cases in blood samples of Ct value below 20, but both were unable to detect malaria cases between 26–40 Ct value ranges amplified by real-time PCR. Despite various diagnostic tools being available, microscopy still remains the method of choice for diagnosis, while the RDT is user-friendly when applied at the point of care. However, our preliminary results emphasize the need to implement the test with higher sensitivity and specificity in the context of a malaria elimination programme. Such programmes can be a crucial opportunity to understand the species prevalent in a low-endemic region. However, these results should be further verified with a large cohort study to document the submicroscopic infection.

## 1. Introduction

Malaria is a vector-borne disease that causes high morbidity and mortality. The five genera of *Plasmodium* that cause malaria fall under the phylum Apicomplexa. Five *Plasmodium* species infect humans: *Plasmodium vivax*, *Plasmodium falciparum*, *Plasmodium malariae*, *Plasmodium ovale*, and *Plasmodium knowlesi* [1]. Around 3.2 billion people in 87 different countries are at risk of importing malaria, and >1 billion are at high risk. According to the World Malaria Report in 2020, there were approximately 229 million cases of malaria worldwide in 2019 and 409,000 deaths. Based on World Health Organization (WHO) reports of malaria, there was a significant drop in malaria cases in 2018, i.e., 535 indigenous cases and 535 imported cases where *Plasmodium vivax* was predominant (85%) [2].

*Plasmodium falciparum* and *Plasmodium vivax* are the predominant species causing malaria in Nepal. *Plasmodium falciparum* is recognized as the most virulent due to its ability to attain high levels of parasitemia via its life cycle, as well as responsible for the majority (91%) of deaths due to major complications from parasite sequestration in deep tissues [3].

Among various conventional diagnostic tools, microscopic examination of Giemsa-stained peripheral blood smears remains the method of choice, but it is dependent upon the parasite load, the course of infection, and on a skilled microscopist, besides that alternative methods for malaria diagnosis such as rapid diagnostic tests (RDTs) are widely available [4]. The majority of the RDT relies on the principle of lateral immunochromatography to detect histidine-rich protein 2 (HRP2), specific to *Plasmodium falciparum* and lactate dehydrogenase or aldolase, enzymes common to all malaria species. The major disadvantages of peripheral blood smear microscopy occur in self-medication without medical consultation, while, in such cases, rapid diagnostic test results remain positive [4, 5]. However, false-positive reactions have been reported with the RDT, due to cross-reaction with rheumatoid factors, and lower sensitivity of RDTs has been reported especially for *Plasmodium ovale*, *Plasmodium vivax*, and *Plasmodium malariae* [6].

Polymerase chain reaction (PCR) methods have been tested for detecting parasites in blood samples with accurate identification of species, both in endemic areas and in countries with imported malaria. The PCR method is reliable and is a confirmatory tool for malaria diagnosis and epidemiological studies in endemic areas due to its high sensitivity and specificity.

The diagnosis of malaria is one of the major concerns in terms of malaria elimination. Laboratory confirmation of malaria prevents unnecessary treatment, development of drug resistance, and rapid correct management of febrile illness [7]. Thus, to improve diagnosis methods, our study aims to compare the performance of microscopy and RDT methods, implementing real-time PCR as the gold standard test in terms of sensitivity and specificity.

## 2. Methodology

### 2.1. Ethical Approval

Ethical clearance was obtained from the Institutional Review Board, BPKIHS. (Ref. No: 257/074/075-IRC (Code No: IRC/1060/017)). Patient consent was obtained by written form, and consent within the pediatric age group was obtained from the patients' guardian.

### 2.2. Study Area and Design

The study was carried out in a tertiary care hospital, B.P. Koirala Institute of Health Sciences (BPKIHS), Dharan (26.80°N, 87.26°E) at province no 1. The total area of BPKIHS is 120000 sq. ft. The region has a tropical climate with two dry seasons (March to May and September to November) and one rainy season (June to August). The mean daily maximum and minimum temperatures are 29–32°C and 17–26°C, respectively. A descriptive cross-sectional study was conducted in a tertiary care hospital in Nepal within the Department of Microbiology between the years 2017 to 2018. This study was conducted in collaboration with the Department of Internal Medicine and Pediatrics. A total of 52 blood samples from cases of acute febrile illness were taken following the convenient sampling technique:

  The inclusion criterion was as follows: patients of all ages visiting the medicine ward, pediatric ward, emergency ward, and the outpatient department with acute febrile illness, i.e., presenting with documented temperatures of ≥37.5°C or a history of fever persisting for 2–7 days
  The exclusion criterion was as follows: patients of all ages visiting the pediatric ward, medicine ward, and the emergency ward other than acute febrile illness cases

### 2.3. Sample Size Determination

The sample size for the study was estimated by using the Haijan-Tilaki method [8] applying the sensitivity of diagnostic tools as follows:

(1) $$\begin{array}{c} N_{\text{SE}}=\left[\frac{Z^{2}\times S_{E}\left(1-S_{E}\right)}{d^{2}\times P}\right], \end{array}$$

where *Z* = 1.96 at 95% confidence level, *S*_*E*_ (sensitivity of standard diagnostic tool) = 0.97, *d*^2^ (margin of error) = 0.05, and *P* (prevalence) = 0.86. The sensitivity of standard diagnostic tools was 97% according to a previous study [9]. The estimated prevalence in the eastern region of Nepal from the previous study was 0.86 [10]. Applying a 95% confidence level value of *Z* = 1.96 and precision or margin of error *d* = 5%. The estimated sample size was calculated as 52.

### 2.4. Sample Collection, Processing, and Storage

Two individual blood samples from patients presenting with documented temperatures of ≥37.5°C (99.5°F) or history of fever persisting for 2–7 days were drawn via venipuncture by an experienced phlebotomist during the period of peak fever, i.e., 38.9–40°C (102–104°F). Sample collection lasted for a period of 6 months, i.e., June 2017 to December 2017.

Blood samples were collected in two EDTA vials: one for peripheral blood smear preparation and RDT analysis, and one for the real-time PCR assay. Blood samples for the PCR assay were stored at −70°C.

### 2.5. Malaria Detection from Blood Films using Light Microscopy

Peripheral blood smears (thick and thin blood films) were prepared and staining was performed using 10% Giemsa solution for 10 minutes. Microscopic examination of Giemsa-stained thick and thin blood smears was performed by observing the minimum 500 oil immersion fields, and this was counterchecked by a medical laboratory technologist and microbiologist with adequate experimental experience [11].

### 2.6. Quantification of Malaria Parasites by the Red Blood Cell (RBC) Count Method using Thin Smear

Parasite quantification was performed using the RBC count method following the WHO protocol [12] using the formula:

(2) $$\begin{array}{c} \text{Estimation of parasitemia }\left(\mu L\right)=\frac{\text{number of parasitized RBC}\left(\text{in}20\text{fields}\right)\times 50000000}{\text{total number of }RBC\text{ count}}. \end{array}$$

The percentage of parasitemia was calculated by counting the infected RBC per 2000 RBCs using the formula:

(3) $$\begin{array}{c} \text{percentage of parasitemia}=\frac{\text{number of Parasitized RBC}\times 100\%}{\text{total number of RBC count}}. \end{array}$$

### 2.7. Malaria Detection using a Rapid Diagnostic Test (RDT)

The histidine-rich protein-2 and *Plasmodium*-specific lactase dehydrogenase–based rapid diagnostic test kit by Malascan™ (lot number 51091) was used [WHO panel detection score: 97% sensitivity for *Plasmodium falciparum* and 90% for *Plasmodium* species other than *Plasmodium falciparum* (PAN) between 2000 and 5000 parasites/*μ*L of the whole blood sample and 82.8% (Pf) and 57.1% (PAN) for 200 parasites/*μ*L of the blood sample with false positivity of 1% for PAN and 0.7% for Pf]. The tests were performed using rapid diagnostic kits, following the manufacturer's instructions [13].

### 2.8. DNA Extraction of Blood Samples using Qiagen Kits

Blood samples, preserved in 0.5 mg EDTA vials, were stored at −70°C to prevent DNA degradation. Samples were left at room temperature for 30 minutes before the extraction process was carried out. DNA was extracted from 200 *μ*L of blood samples using QIAamp DNA blood minikits (Qiagen, Hilden, Germany, catalog number 51104) following the manufacturer's instructions in the QIAamp DNA mini-handbook [14].

#### 2.8.1. Taqman Assay-Based Real-Time PCR for Amplification of Different *Plasmodium* Species

Extracted DNA was amplified using a Qiagen Rotor-Gene Q system using abTes™ Malaria 5 qPCR commercially available kit (catalog number 300229) using a set of specific primers for each *Plasmodium* species designed by the manufacturer. Firstly, a master mix solution was prepared using the following scheme: primer/probe: 2 *μ*L, enzyme/reaction mix: 6 *μ*L, nuclease-free water: 12 *μ*L, and template: 5 *μ*L. The thermal cycler was programmed as phase 1 at 95°C for 2 min to implement one cycle, phase 2 at 60°C for 20 seconds to produce 30 cycles, and phase 3 at 68°C for 1 minute per kb to generate 30 cycles on the basis of manufacturer's instructions. Fluorescence intensity was measured using the Rotor gene Q software, and the graph of fluorescence intensity vs. the number of cycles was plotted [15].

Real-time PCR-based TaqMan assay was performed to identify the species and compare the results with microscopy and the RDT. *Plasmodium* DNA was amplified. Five fluorescent dyes, i.e., FAM, HEX, ROX, Cy5, and Quasar 705, were used to detect five different species of *Plasmodium* [15], i.e.:

  FAM (green)-*Plasmodium falciparum*,
  Cy5 (red)-*Plasmodium ovale*
  HEX (yellow)-*Plasmodium malariae*,
  ROX (orange)-*Plasmodium vivax*
  Quasar 705(crimson)-*Plasmodium knowlesi*

where positive control = all malaria-positive control available in the commercial kit and negative control = phosphate buffer saline.

Blood samples were considered positive for malaria based on fluorescence intensities which were greater than the threshold or cutoff values.

### 2.9. Data Management and Statistical Analysis

The collected data were entered into an excel spreadsheet, coding was done for different variables, and the data was transferred to SPSS version 11.5. The comparison between different diagnostic tools was done using real-time PCR as the gold standard assay. Applying the chi-square test by constructing 2 × 2 table, sensitivity, specificity, positive predictive value, negative predictive value, and accuracy of the diagnostic tests were calculated. The level of agreement between microscopy and the RDT were estimated by using the interrelated kappa coefficient, and *p* values were calculated by using the chi-square test by using SPSS [16].

## 3. Results

### 3.1. Demographics of the Study

**Figure 1:**
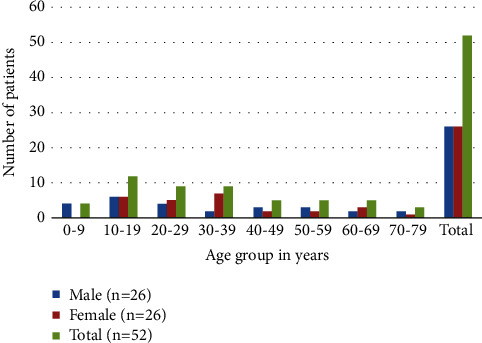
Demographic classification of gender based on different age groups.

In our study, 52 individuals were involved, with an equal number (*n* = 26) of men and women (Figure 1). The highest number of participants were from the 10–19 (*n* = 12) age group. The fewest participants were in the age group 70–79.

### 3.2. Microscopic Examination of Peripheral Blood Smears

**Figure 2:**
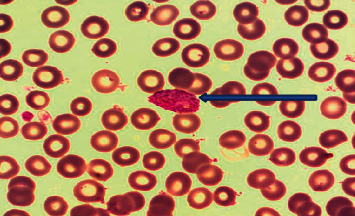
Schizont of *Plasmodium vivax*.

**Figure 3:**
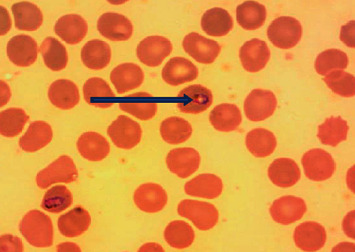
Ring trophozoites of *Plasmodium falciparum*.

Out of the total of 52 whole blood samples, *n* = 3 (5.8%) samples were positive by microscopic examination, and *n* = 49 (94%) samples were negative by microscopic findings. Schizonts of *Plasmodium vivax* (Figure 2) and ring trophozoites of *Plasmodium falciparum* (Figure 3) were detected by light microscopy in the peripheral blood smears.

*Plasmodium* species detected using light microscopy were quantified using the RBC count method from thin blood films (Table 1).

Plasmodium species identified by light microscopy was quantified from a thin smear following the protocols of WHO guidelines in which the highest parasites/*μ*L was 12000 parasites and the lowest was 7000 parasites in our study amongst three malaria positive cases identified by light microscopy.

### 3.3. Rapid Diagnostic Test Results

Out of the total 52 blood samples, *n* = 7(13.5%) blood samples were positive for malaria where *n* = 3 (5.8%) were found to be *Plasmodium falciparum*, *n* = 4 (7.69%) were found to be species other than *Plasmodium falciparum*, and *n* = 45(87%) cases were negative using immunochromatography tests.

### 3.4. Taqman Assay-Based Real-Time PCR Results

**Figure 4:**
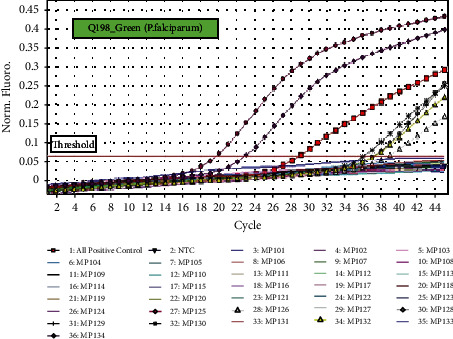
qPCR amplification of blood samples infected by *Plasmodium falciparum*.

**Figure 5:**
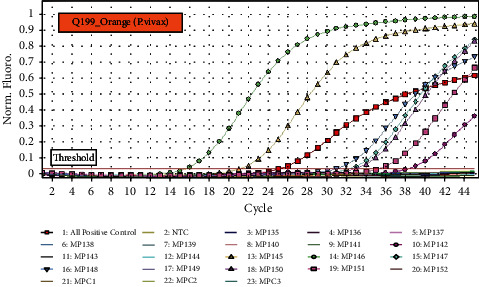
qPCR amplification of blood samples infected by *Plasmodium vivax*. All Malaria-positive control = positive control provided in a commercial kit. NTC = negative test control (phosphate buffer saline).

Out of the total 52 whole blood samples, *n* = 14(27%) blood samples were positive for the detection of *Plasmodium* species and *n* = 38(73%) blood samples were negative for *Plasmodium* species using real-time PCR assay. Amplification of *Plasmodium* DNA from 52 blood samples of patients with acute febrile illness is shown in Figures 4 and 5 along with all malaria-positive controls and negative test controls (NTC).

### 3.5. Prevalence of Diagnosed Malaria Cases by Various Diagnostic Methods

The detection of malaria from whole blood samples were implemented by using three diagnostic tools such as light microscopy, RDT, and real-time PCR in our study. The prevalence of cases stratified by microscopy was 5.8%, by the RDT was 13.5%, and by real-time PCR was 27%. Large proportions of cases were missed by light microscopy (94%) and the RDT (86.5%). Unfortunately, *n* = 3 (5.8%) were falsely diagnosed by the RDT for malaria.

### 3.6. Comparison of Diagnostic Accuracy of Light Microscopy and the RDT vs. PCR

Using real-time PCR as a reference method, 14 (27%) participants were positive for malaria while 38 (73%) participants were negative (Table 2). The RDT correctly identified 4 (7.69%) participants, whereas microscopy accurately identified 3 (5.8%) participants (true positive). The sensitivity of light microscopy and the RDT was 21.4% and 28.6%, respectively; the specificity of light microscopy and the RDT was 100% and 92.1%, respectively; and the accuracy of light microscopy and the RDT was 78.84% and 75%, respectively. Light microscopy (kappa 0.285) and RDT (kappa 0.246) had a fair agreement when compared to real-time PCR. In general, the positive predictive value of microscopy and the RDT was 100% and 57.14%, whereas the negative predictive value of microscopy and the RDT was 77.6% and 77.8%. Although the performance of microscopy was far less compared to PCR, microscopy yielded 100% specificity among the identified species and was statistically significant (*p* value <0.05).

### 3.7. Comparison of Microscopy and RDT Results with the Cycle Threshold (Ct) Value of Real-Time PCR

**Figure 6:**
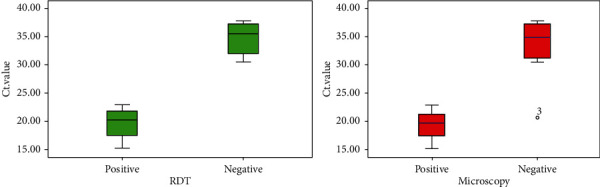
Box plots diagram representing RDT and microscopy results with the Ct value of real-time PCR.

Microscopy and the RDT had a similar rate of detection as PCR for malaria-positive cases with a Ct value below 20. Microscopy diagnosis reduced to 50% as it failed to detect one positive malaria case between 20 and 25 Ct value (Figure 6). However, RDT was found to be more effective compared to microscopy because of its potential to estimate malaria cases with the same accuracy as real-time PCR between 20 and 25 Ct value (Table 3). From our study results, it was marked that the RDT and microscopy together were unable to detect malaria cases between 26 and 40 Ct value ranges. Nevertheless, the RDT was found to be sensitive compared to microscopy in malaria diagnosis. Based on comparison between the mean Ct value of PCR with microscopy and the RDT among positive cases, the significant difference (*p* < 0.05) in mean Ct value was observed when comparison was done individually by applying Student's *t*-test as summarized below in Table 3:

## 4. Discussion

Accurate estimation of malaria burden is essential for malaria management following a prompt treatment regimen. The diagnosis of malaria has been a remarkable challenge, especially in poor regions, due to the availability of limited resources, equipment, and methods. Laboratory diagnostic methods have been mandatory for the detection of cases and the timely initiation of appropriate therapy. The study was conducted to determine the proportion of malaria cases missed by microscopy and the RDT but detected by real-time PCR. Microscopy and rapid diagnostic tests are routinely used diagnostic tools. Research in other countries suggests a fivefold difference of estimated prevalence using light microscopy and PCR [17, 18]. In many parts of Nepal and other malaria-endemic developing countries, light microscopy is still used as the standard tool due to the limited supply and limited consistency of the supply of RDTs [19–21]. In this study, peripheral blood smear (PBS) missed 78.57% of malaria when compared with real-time PCR. Previous meta-analysis data from 42 studies determined that microscopy missed about 50% of PCR-positive malaria infections [17]. Moreover, in a large epidemiological study in Cambodia, a large proportion of malaria-missed samples by light microscopy were successfully detected by PCR (289/7491; 3.85%) [22].

Other studies in Southeast Asia have reported similar results where light microscopy has detected a low level of malaria [17, 18]. The study conducted in Myanmar showed the rate of detection of malaria using real-time PCR was higher than light microscopy and the RDT with 100% sensitivity and specificity [23]. A false-negative microscopy result is directly proportional to the decrease in parasite density due to its limit of detection (LOD) about 50 parasites/*μ*l of blood [13, 24, 25]. The LOD varies from 30–100 parasites/*μ*L between expert and field microscopists. This implies that the negative results of microscopy does not exclude *Plasmodium* infection. The RDT missed 71% of real-time PCR-positive infections. However, the sensitivity of malaria detection using the RDT is greater than that of microscopy, which is consistent with the study conducted in Kenya [26]. Limitations of RDT include false positivity due to cross-reaction and LOD ranges from 50–100 parasites/*μ*L [6, 25, 27]. We observed 5.76% of false-positive cases when compared to those of real-time PCR. In a research study conducted in Australia comparing different RDT methods, the false positive rate was found to be 8.33% that supports our findings [25]. Alternative study in Equatorial Guinea recorded 13.3% of false-positive cases of the RDT [27] due to cross-reaction of rheumatoid factors or residues of HRP2 antigen circulating in the blood after parasite clearance [6, 27]. RDT kits applied in our study distinguished only a single species, i.e., *Plasmodium falciparum*. This was due to the merged pLDH panels for different *Plasmodium* species. RDT's sensitivity was 28.6% with a specificity of 92% in our experiment. Other studies showed RDT sensitivities ranging from 60 to 80% and specificities between 80 and 90% based on the detection of different *Plasmodium species* [5, 25, 28]. A study in Tanzania also reported 0.92% of false-positive cases by the RDT [29]. Our study concluded that submicroscopic infections missed by RDTs and peripheral blood smears were successfully detected by real-time PCR. Identical results were observed in Equatorial Guinea's experiment where 19.4% of samples were false negative by microscopy and 13.3% were false negative by the RDT [28]. Other previous studies also support missed cases of submicroscopic infection by microscopy and RDTs [6]. Microscopic examination in our study had a low sensitivity (21.4%) and a high specificity (100%) with an accuracy of 78.84%. Different broad studies found lower sensitivity of light microscopy compared to that of molecular tools, and relatively good specificity of light microscopy in many endemic fields [30–32]. Few reported cases of false diagnosis using light microscopy. One instance was from Myanmar in misinterpretation of *Plasmodium falciparum* with *Plasmodium vivax* that was later confirmed by PCR [33].

Real-time PCR performance showed better sensitivity and specificity than conventional PCR in several past research studies [31, 34, 35]. An earlier study conducted by Gatti et al. reported that conventional PCR failed to detect mixed infections which were detected by microscopy [36]. A PCR study in *Plasmodium* species conducted in the US showed real-time PCR was a reliable confirmatory tool for the diagnosis of malaria to resolve conflicting doubts between light microscopy and RDT [34]. A research work in Nigeria found the sensitivity and specificity of PCR to be 100%, whereas 58%–60% of the cases were falsely diagnosed by microscopy and RDT [37]. False diagnosis by light microscopy happens due to several factors based on experience as well as improper reagent quality [9, 38].

Our PCR data helped us to understand the difference between light microscopy and RDT results. The LOD of the RDT was roughly comparable to light microscopy in the field survey, although the newer generation of RDTs showed a higher sensitivity than the previous ones [5, 6, 39]. Moreover, the RDT performed better than light microscopy in surveys demonstrated by various studies [29, 31]. The use of qPCR in our study increased the prevalence of malaria approximately twofold in comparison with the use of the RDT and greater than fourfold compared to the use of microscopy, and this is similar to the differences between PCR- and RDT-detected prevalence reported elsewhere [6, 40]. The Ct value of malaria positive samples estimated by real-time PCR helped us to determine the performance of light microscopy and RDT. Light microscopy and the RDT showed better efficacy on malaria-positive samples of Ct value <20. RDT did not miss any cases even on positive samples of Ct values ranged between 20 and 25, whereas microscopy missed a positive malaria case between 20 and 25 Ct value. Both conventional diagnostic tools (microscopy and RDT) were unable to detect malaria cases on positive samples >25 Ct value estimated by real-time PCR. The study conducted in Norway revealed the low efficiency of light microscopy for malaria-positive samples of Ct value >20 as well as both microscopy and the RDT failed to detect malaria in blood samples with low parasitemia (Ct value >30) that showed similarity with our findings [41].

Light microscopy efficiency seems inadequate for surveillance of parasite infections in Nepal in areas where the intensity of transmission rate is shifting from high to low. The RDT test has the advantage of allowing empirical treatment for symptomatic individuals with positive RDT results. Our preliminary results emphasized the need to implement the test with the higher sensitivity and specificity in the context of a malaria elimination programme, which provides an opportunity to understand the parasite circulation in a low-endemic region. However, these results should be further verified with a large cohort study to document the submicroscopic infection.

On examining the cost analysis of each methodology applied in our study, one of the major challenges in a low-income country for the diagnosis of malaria and conducting a large cohort study is a cost barrier in different applied diagnostic methodology. In addition, the RDT costs (11.25 USD per single test) are more expensive than the routine investigation of peripheral blood smear microscopy (4 USD per test, excluding the cost of materials such as a microscope, latex gloves, reagents, and a skilled microscopist). DNA extraction kits per box (500 USD) cost approximately 60000 NPR (500 USD) and master mix reagent kits also cost approximately 500 USD per box (50 test) in Nepal (additional cost includes powder-free nitrile gloves costing 16 USD per box and series of micropipettes with different pipetting capacities costs approximately 50 USD per pipette) [42]. The cost of expertise manpower varies based on the type of health care sector. Due to the financial issues in resource-limited settings, there are remarkable challenges in the laboratory diagnosis of malaria where the transmission rate is shifting from high to low due to an increase in trends of submicroscopic infections, our findings encourage the application of microscopy and the RDT along with the real-time PCR to track, test, and treat malaria cases throughout the region.

## 5. Conclusions

Although light microscopy remains a standard method for detecting malaria parasites, it has many shortcomings despite the use of experienced microscopists, such as the quality control of reagents, effects of self-medication, and the inability to detect low levels of parasitemia. This implies that the presence of a large proportion of submicroscopic parasitemia at tertiary care hospitals has been vastly underestimated. The application of PCR adds to our understanding of the malaria transmission level, in the sense that they can determine whether recently cleared infections are treated or not and are no longer detectable by a light microscope and the RDT. Thus, applying all available diagnostic tools (i.e., PCR, RDT, and light microscopy) together was able to detect actual parasitemia and recent infections and may provide the most precise information to decide on the best malaria control strategies.

## Figures and Tables

**Table 1:** Quantification of parasitemia using the RBC count method among *Plasmodium* species identified in the blood film using light microscopy.

Sample code	Microscopic examination	Infected RBCs per 20 fields (100*x*)	Parasites(per *μ*L)	Infected RBCs per 2000 RBCs	Parasitemia (%)
MP125	Trophozoites of *Plasmodium falciparum*	12	12000	5	0.25
MP134	Trophozoites of *Plasmodium falciparum*	10	10000	4	0.2
MP146	Schizonts of *Plasmodium vivax*	7	7000	3	0.15

**Table 2:** Performance characteristics of microscopy and RDT when compared with real-time PCR.

Test characteristics	Microscopic examination	RDT
TP (PCR = 14)	3	4
FP (PCR negative)	0	3
TN (PCR = 38)	38	35
FN (PCR positive)	11	10
Sensitivity	21.4%	28.6%
Specificity	100%	92.1%
PPV	100%	57.14%
NPV	77.6%	77.8%
Accuracy	78.84%	75%
Kappa value	0.285	0.246
*p* value	0.003	0.053

TP = true positive, FP = false positive, TN = true negative, FN = false negative, PPV = positive predictive value, and NPV = negative predictive value.

**Table 3:** Summary table representing the comparison of real-time PCR cycle threshold (Ct) value with the positive results of microscopy and the RDT.

Ct value (ranges)	Microscopy detection rate	RDT detection rate
<20 (*n* = 2)	2 (100%)	2 (100%)
20–25 (*n* = 2)	1 (50%)	2 (100%)
26–30 (*n* = 1)	Not detected	Not detected
31–35 (*n* = 2)	Not detected	Not detected
36–40 (*n* = 6)	Not detected	Not detected
Mean Ct value ± standard deviation 30.30 ± 7.6	18.31 ± 3.86 (*p* value = 0.029)	19.66 ± 2.03 (*p* value = 0.016)

## Data Availability

Most of the data are included in our study. However, detailed data of the experiment can be obtained upon request from the corresponding author.

## Abbreviations

Pf: *Plasmodium falciparum*
PAN: Species other than *Plasmodium falciparum*
RDT: Rapid diagnostic test
PCR: Polymerase chain reaction
qPCR: Quantitative PCR
HRP: Histidine-rich protein
PPV: Positive predictive value
NPV: Negative predictive value.

## Additional Points

Due to the lack of available and limited resources as well as funding in a low-income country, large cohort study could not be conducted and other molecular methods could not be run simultaneously with PCR to estimate submicroscopic infections of malaria that were missed by microscopy and the RDT.

## Supplementary Materials

Supplementary Materials The first-uploaded (20180726_qPCR_Experiment No Q199.pdf) and second-uploaded supplementary files (20180726_qPCR_Experiment No Q198.pdf) were the sheets generated by Rotor-Gene Q PCR machine that shows various chemical reagents used in our PCR experiments, PCR master mix components with their final volumes, thermocycling conditions (temperature, time, cycles, and optics), and real-time PCR graphical analysis for the detection of all five *Plasmodium* species along with their positive and negative controls. The two detected *Plasmodium* species in the whole blood samples (*Plasmodium falciparum* and *Plasmodium vivax*) amplified by real-time PCR were represented along with their Ct values.
