# Supplementary material for: Challenges in Laboratory Diagnosis of Malaria in a Low-Resource Country at Tertiary Care in Eastern Nepal: A Comparative Study of Conventional vs. Molecular Methodologies
Source: J Trop Med. 2021 Dec 28;2021:3811318. doi: 10.1155/2021/3811318 (PMC8727160; doi:10.1155/2021/3811318)
Supplement: Supplementary Materials — The first-uploaded (20180726_qPCR_Experiment No Q199.pdf) and second-uploaded supplementary files (20180726_qPCR_Experiment No Q198.pdf) were the sheets generated by Rotor-Gene Q PCR machine that shows various chemical reagents used in our PCR experiments, PCR master mix components with their final volumes, thermocycling conditions (temperature, time, cycles, and optics), and real-time PCR graphical analysis for the detection of all five Plasmodium species along with their positive and negative controls. The two detected Plasmodium species in the whole blood samples (Plasmodium falciparum and Plasmodium vivax) amplified by real-time PCR were represented along with their Ct values. [file 3811318.f1.zip › 3811318.f1/20180726_qPCR_Experiment No Q198.pdf]

Molecular Biology Laboratory

BP Koirala Institute of Health Science

RGQ Machine Nr RGQ_02

qPCR Experiment No: Q198

Date : 26/07/2018

PCR Name **Malaria 5 qPCR**

SOP No. MLB_BP_0062_1.1

1. To perform the Malaria multiplex PCR of clinical sample

2. Required Material:

SN	Chemical /Reagents	Brand	Catalogue Number	Lot Number
1	PCR grade water	Himedia	ML024	0000117943
2	Primer/Probe Mix	AIT biotech	300230	E229-10
3	Enzyme/Reaction Mix	AIT biotech	300230	E229-10
4	Positive Control	AIT biotech	300230	E229-10
	**Number of reaction tubes**	37		

SN	RXN components	Stock Concentration	Final concentration	Volumes/ tube (µl)	Final volume (µl)
1	Primer/Probe Mix	NA	NA	2	74
2	Enzyme/Reaction Mix	NA	NA	6	222
15	Water Nuclease free	NA	NA	12	444
16	DNA Template			5	
	Total			25	740

Aliquot 20µl of master mix in each 36 Tubes: 34 Samples + 1 Positive Controls + 1 No template control (NTC)

3. Name of Rotor Gene Q: Rotatory ramp, Convectional Heating

4. Programme Condition:

Temp (˚C)	Time		Cycles	Optics
**95**	2	min	1	Off
**95**	5	sec	45	Off
**60**	20	sec		On

5. qPCR Analysis:

Location of the Folder on Computer: D:\Molecular Lab\Google_drive\qPCR\

Name of the RGQ file: Q198_Malaria_qPCR_V1 2018-07-26

Tube No	Sample code	ct VALUE					PCR RESULT	Malaria Species
		FAM (*P.falciparum*)	HEX (*P.malariae*)	ROX (*P.vivax*)	Cy5 (*P.ovale*)	Quasar 705 (*P.knowlesi*)		
1	All Positive Control	28.88	30.03	30.57	29.96	28.11	POSITIVE	
2	No template Control						NEGATIVE	
3	MP101						NEGATIVE	
4	MP102						NEGATIVE	
5	MP103						NEGATIVE	
6	MP104						NEGATIVE	
7	MP105						NEGATIVE	
8	MP106						NEGATIVE	
9	MP107						NEGATIVE	
10	MP108						NEGATIVE	
11	MP109						NEGATIVE	
12	MP110						NEGATIVE	
13	MP111						NEGATIVE	
14	MP112						NEGATIVE	
15	MP113						NEGATIVE	
16	MP114						NEGATIVE	
17	MP115						NEGATIVE	
18	MP116						NEGATIVE	
19	MP117						NEGATIVE	
20	MP118						NEGATIVE	
21	MP119						NEGATIVE	
22	MP120						NEGATIVE	
23	MP121						NEGATIVE	
24	MP122						NEGATIVE	
25	MP123						NEGATIVE	
26	MP124						NEGATIVE	
27	MP125	19.76					POSITIVE	*P.falciparum*
28	MP126	38.86					POSITIVE	*P.falciparum*
29	MP127						NEGATIVE	
30	MP128	37.31					POSITIVE	*P.falciparum*
31	MP129	37.27					POSITIVE	*P.falciparum*
32	MP130	36.19					POSITIVE	*P.falciparum*
33	MP131						NEGATIVE	
34	MP132	37.36					POSITIVE	*P.falciparum*
35	MP133						NEGATIVE	
36	MP134	22.94					POSITIVE	*P.falciparum*

Green Channe

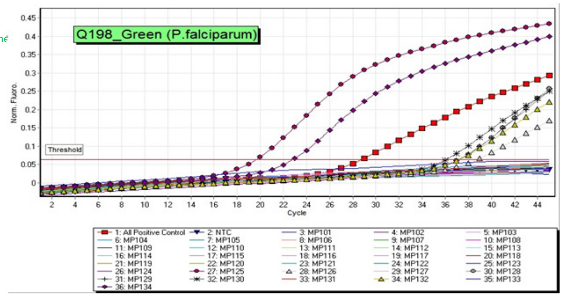

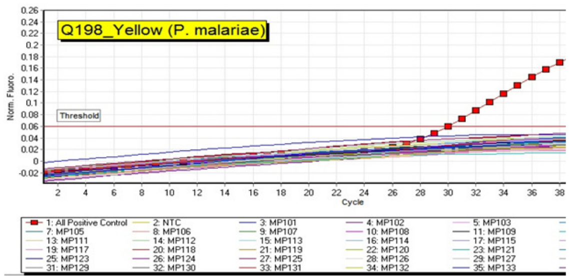

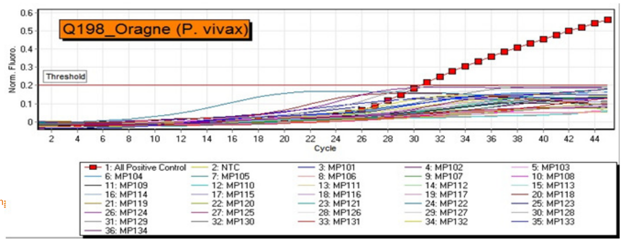

Oran

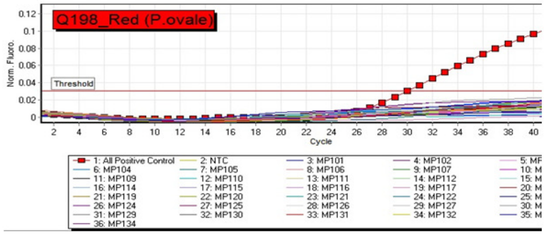

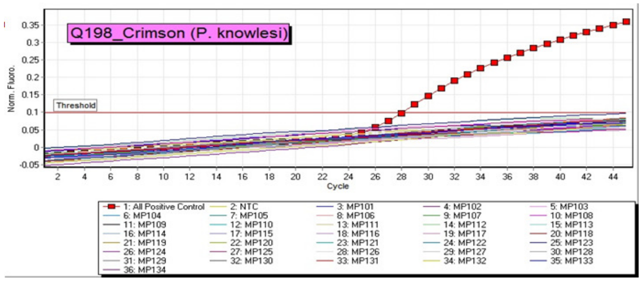

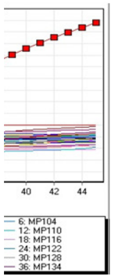
40
42
44
6: MP104
12: MP110
18: MP116
24: MP122
30: MP128
36: MP134

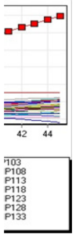
42
44
P103
P108
P113
P118
P123
P128
P133
