# Supplementary material for: Challenges in Laboratory Diagnosis of Malaria in a Low-Resource Country at Tertiary Care in Eastern Nepal: A Comparative Study of Conventional vs. Molecular Methodologies
Source: J Trop Med. 2021 Dec 28;2021:3811318. doi: 10.1155/2021/3811318 (PMC8727160; doi:10.1155/2021/3811318)

Molecular Biology Laboratory

BP Koirala Institute of Health Science

RGQ Machine Nr RGQ_01

qPCR Experiment No: Q199

Date : 26/07/2018

PCR Name **Malaria 5 qPCR**

SOP No. MLB_BP_0062_1.1

1. To perform the Malaria multiplex PCR of clinical sample

2. Required Material:

SN	Chemical /Reagents	Brand	Catalogue Number	Lot Number
1	PCR grade water	Himedia	ML024	0000117943
2	Primer/Probe Mix	AIT biotech	300230	E229-10
3	Enzyme/Reaction Mix	AIT biotech	300230	E229-10
4	Positive Control	AIT biotech	300230	E229-10
	**Number of reaction tubes**	24		

SN	RXN components	Stock Concentration	Final concentration	Volumes/ tube (µl)	Final volume (µl)
1	Primer/Probe Mix	NA	NA	2	48
2	Enzyme/Reaction Mix	NA	NA	6	144
15	Water Nuclease free	NA	NA	12	288
16	DNA Template			5	
	Total			25	480

Aliquot 20µl of master mix in each 23 Tubes: 21 Samples + 1 Positive Controls + 1 No template control (NTC)

3. Name of Rotor Gene Q: Rotatory ramp, Convectional Heating

4. Programme Condition:

Temp (˚C)	Time		Cycles	Optics
**95**	2	min	1	Off
**95**	5	sec	45	Off
**60**	20	sec		On

5. qPCR Analysis:

Location of the Folder on Computer: D:\Molecular Lab\Google_drive\qPCR\

Name of the RGQ file: Q199_Malaria_qPCR_V1 2018-07-26

		ct VALUE						
**Tube No**	**Sample code**	**FAM (*P.falciparum*)**	**HEX (*P.malariae*)**	**ROX (*P.vivax*)**	**Cy5 (*P.ovale*)**	**Quasar 705 (*P.knowlesi*)**	**PCR RESULT**	**Malaria Species**
1	All Positive Control	28.01	29.09	24.69	28.85	24.71	POSITIVE	**Pos control**
2	No template Control						NEGATIVE	
3	MP135						NEGATIVE	
4	MP136						NEGATIVE	
5	MP137						NEGATIVE	
6	MP138						NEGATIVE	
7	MP139						NEGATIVE	
8	MP140						NEGATIVE	
9	MP141						NEGATIVE	
10	MP142			37.81			POSITIVE	***P.vivax***
11	MP143						NEGATIVE	
12	MP144						NEGATIVE	
13	MP145			20.71			POSITIVE	***P.vivax***
14	MP146			15.24			POSITIVE	***P.vivax***
15	MP147			32			POSITIVE	***P.vivax***
16	MP148			30.53			POSITIVE	***P.vivax***
17	MP149						NEGATIVE	
18	MP150			32.56			POSITIVE	***P.vivax***
19	MP151			34.91			POSITIVE	***P.vivax***
20	MP152						NEGATIVE	
21	MPC1						NEGATIVE	
22	MPC2						NEGATIVE	
23	MPC3						NEGATIVE	

Gree

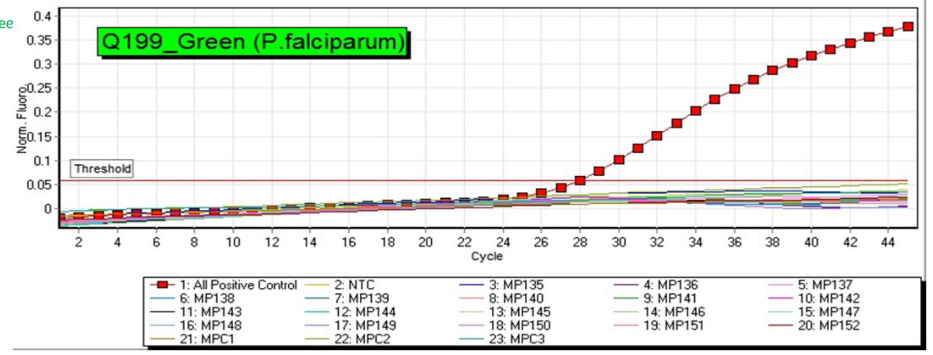

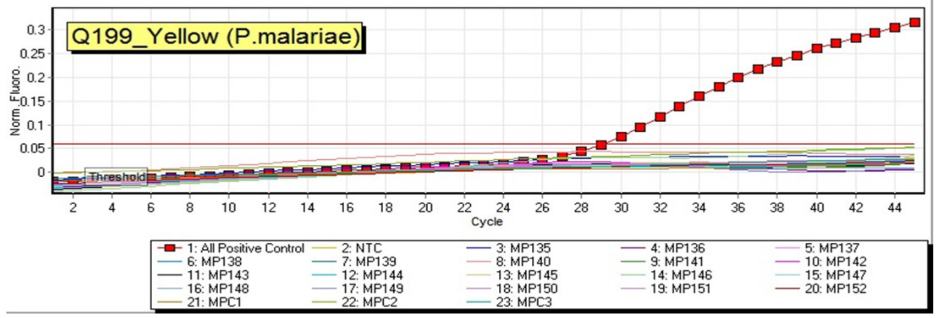

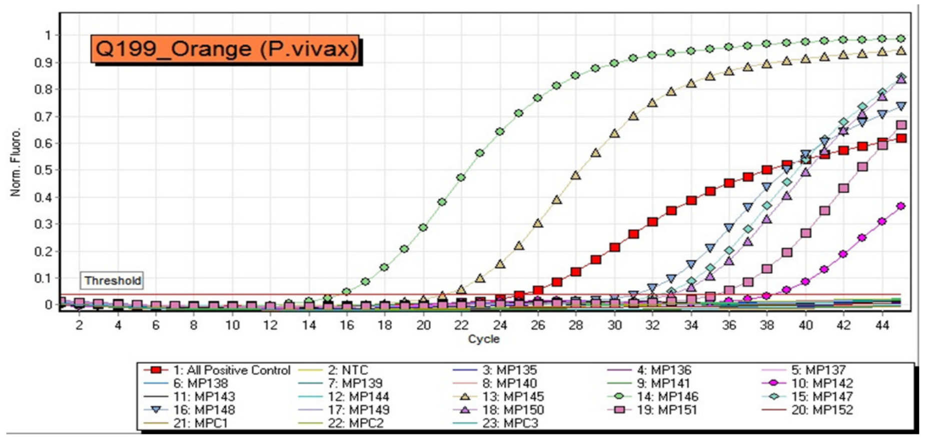

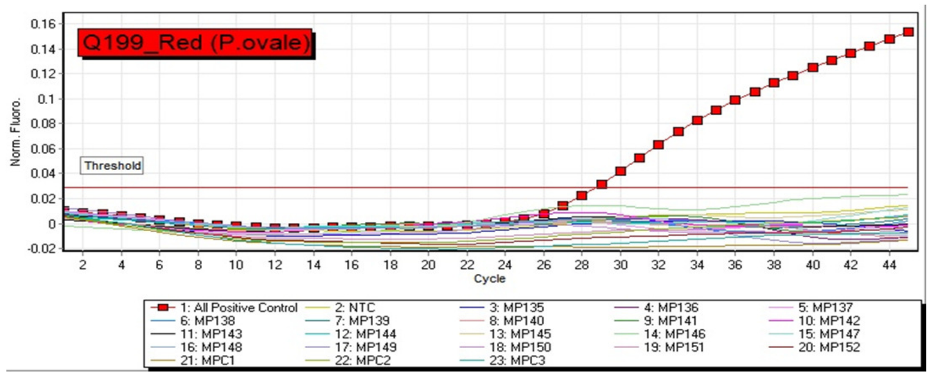

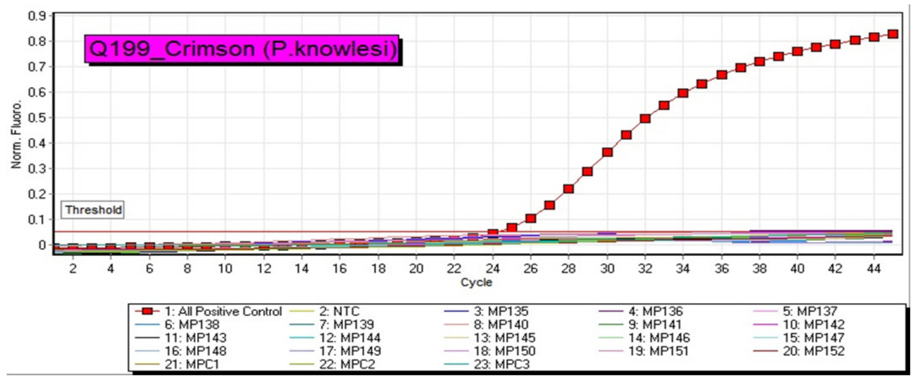

Supplement: Supplementary Materials — The first-uploaded (20180726_qPCR_Experiment No Q199.pdf) and second-uploaded supplementary files (20180726_qPCR_Experiment No Q198.pdf) were the sheets generated by Rotor-Gene Q PCR machine that shows various chemical reagents used in our PCR experiments, PCR master mix components with their final volumes, thermocycling conditions (temperature, time, cycles, and optics), and real-time PCR graphical analysis for the detection of all five Plasmodium species along with their positive and negative controls. The two detected Plasmodium species in the whole blood samples (Plasmodium falciparum and Plasmodium vivax) amplified by real-time PCR were represented along with their Ct values. [file 3811318.f1.zip › 3811318.f1/20180726_qPCR_Experiment No Q199.pdf]
